# Phenotypic and genotypic characterization of families with complex intellectual disability identified pathogenic genetic variations in known and novel disease genes

**DOI:** 10.1038/s41598-020-57929-4

**Published:** 2020-01-22

**Authors:** Hossein Darvish, Luis J. Azcona, Abbas Tafakhori, Roxana Mesias, Azadeh Ahmadifard, Elena Sanchez, Arman Habibi, Elham Alehabib, Amir Hossein Johari, Babak Emamalizadeh, Faezeh Jamali, Marjan Chapi, Javad Jamshidi, Yuji Kajiwara, Coro Paisán-Ruiz

**Affiliations:** 1 Cancer Research Center, Semnan University of Medical Sciences, Semnan, Iran; 2 Department of Medical Genetics, Semnan University of Medical Sciences, Semnan, Iran; 3 Department of Neuroscience, Icahn School of Medicine at Mount Sinai, One Gustave L. Levy Place, New York, NY 10029 USA; 4 Department of Neurology, Icahn School of Medicine at Mount Sinai, One Gustave L. Levy Place, New York, NY 10029 USA; 5 Iranian Center of Neurological Research, Neuroscience Institute, Tehran University of Medical Sciences, Tehran, Iran; 6 The Graduate School of Biomedical Sciences, Icahn School of Medicine at Mount Sinai, One Gustave L. Levy Place, New York, NY 10029 USA; 7 Student Research Committee, School of Medicine, Shahid Beheshti University of Medical Sciences, Tehran, Iran; 8 Department of Medical Genetics, Faculty of Medicine, Tabriz University of Medical Sciences, Tabriz, Iran; 9 Noncommunicable Diseases Research Centre, Fasa University of Medical Sciences, Fasa, Iran; 10 Neuroscience Research Australia, Randwick, Sydney, NSW Australia; 11 Denali Therapeutics, 161 Oyster Point Blvd, South San Francisco, CA 94080 USA; 12 Department of Psychiatry, Icahn School of Medicine at Mount Sinai, One Gustave L. Levy Place, New York, NY 10029 USA; 13 Department of Genetics and Genomic sciences, Icahn School of Medicine at Mount Sinai, One Gustave L. Levy Place, New York, NY 10029 USA; 14 Mindich Child Health and Development Institute, Icahn School of Medicine at Mount Sinai, One Gustave L. Levy Place, New York, NY 10029 USA; 15 Friedman Brain Institute, Icahn School of Medicine at Mount Sinai, One Gustave L. Levy Place, New York, NY 10029 USA

**Keywords:** Genetic linkage study, Neurodevelopmental disorders

## Abstract

Intellectual disability (ID), which presents itself during childhood, belongs to a group of neurodevelopmental disorders (NDDs) that are clinically widely heterogeneous and highly heritable, often being caused by single gene defects. Indeed, NDDs can be attributed to mutations at over 1000 loci, and all type of mutations, ranging from single nucleotide variations (SNVs) to large, complex copy number variations (CNVs), have been reported in patients with ID and other related NDDs. In this study, we recruited seven different recessive NDD families with comorbidities to perform a detailed clinical characterization and a complete genomic analysis that consisted of a combination of high throughput SNP-based genotyping and whole-genome sequencing (WGS). Different disease-associated loci and pathogenic gene mutations were identified in each family, including known (n = 4) and novel (n = 2) mutations in known genes (*NAGLU*, *SLC5A2*, *POLR3B*, *VPS13A*, *SYN1*, *SPG11*), and the identification of a novel disease gene (n = 1; *NSL1*). Functional analyses were additionally performed in a gene associated with autism-like symptoms and epileptic seizures for further proof of pathogenicity. Lastly, detailed genotype-phenotype correlations were carried out to assist with the diagnosis of prospective families and to determine genomic variation with clinical relevance. We concluded that the combination of linkage analyses and WGS to search for disease genes still remains a fruitful strategy for complex diseases with a variety of mutated genes and heterogeneous phenotypic manifestations, allowing for the identification of novel mutations, genes, and phenotypes, and leading to improvements in both diagnostic strategies and functional characterization of disease mechanisms.

## Introduction

Intellectual disability (ID) is a neurodevelopmental disorder (NDD) characterized by significantly impaired intellectual and adaptive function. ID affects about 2–3% of the general population, with the majority of the cases (75–90%) suffering from mild ID and with only 10–25% of the cases reporting moderate or severe ID^1^. It is subdivided into two subtypes, known as non-syndromic ID (NSID), in which cognitive impairment is the only apparent clinical symptom, as further phenotyping (MRI, biochemical profiling, and so on) often reveals additional symptoms, and syndromic ID (SID), in which intellectual deficits are accompanied by other neurological and behavioral manifestations. ID is also frequently seen in autistic children, who exhibit deficits in social communication and interaction, repetitive behaviors, and/or restricted interests^2–4^.

The majority of ID disorders are caused by single genetic defects^5^, the discovery of which is vastly accelerating due to advances in genomics and high throughput sequencing. High throughput sequencing in Autism Spectrum Disorders (ASD) has led to the identification of a large number of rare variations in both DNA sequence and chromosomal structure conferring disease risk^6,7^. Given the large number of genes and genetic variations implicated in NDD syndromes, a thorough screening of the genome is the most suitable molecular technique to identify the disease-causing genomic events.

In this study, we examined seven different families featuring complex NDD syndromes, in which ID was accompanied by other neurodegenerative and/or metabolic manifestations, and performed homozygosity mapping (HM) through high throughput SNP genotyping and WGS to identify the disease-associated loci and pathogenic and genetic variations. All but one of the examined pedigrees belong to consanguineous recessive marriages, in which the affected subjects, due to identity by descent, are likely to have two identical copies of the disease allele derived from a common ancestor; thus, reducing genetic heterogeneity. Herein, the use of HM to discover disease-associated loci and reduce variant screening in subsequent WGS analyses is particularly powerful in these families. We identified pathogenic variations in seven different disease genes and carried out functional assays in one disease gene associated with both ID and autistic-like symptoms. Detailed clinical characterization was also performed in family with the objective to assist with the genotype-phenotype correlations and clinical diagnoses of prospective families.

## Results

### Clinical findings

**Figure 1:**
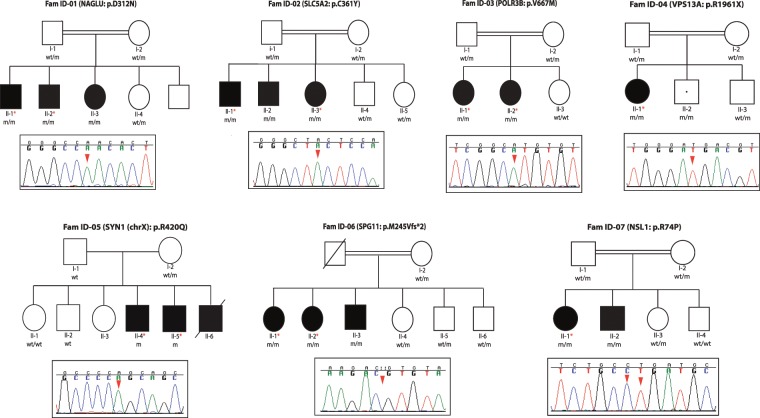
Pedigree structures of families presenting with ID syndromes and their corresponding pathogenic mutations. The pedigrees of seven families featuring NDD syndromes are shown. Affected members are represented by either dark squares (males) or circles (females). The only individual who is non-manifesting for the phenotype but is homozygous for the mutation is represented with a white square with a black dot in the middle. Individuals with homozygous mutations are represented as m/m; heterozygous carriers as wt/m; and non-carriers individuals homozygous for the healthy allele as wt/wt. *Indicates those individuals that were subject to WGS analyses. The *Sanger* chromatogram sequences of the identified pathogenic mutations highlighting the homozygous mutant alleles (red arrow) are shown below each pedigree.

We recruited and clinically characterized seven different families featuring complex NDD disorders (Fig. 1A). The detailed clinical descriptions for each individual family are compiled in Table 1. Briefly, five families presented with ID and movement disorder phenotypes, including ataxia, spastic paraplegia, and chorea-acanthocytosis, among others, and two families presented with severe ID that was accompanied with autistic-like features as well as language behaviors in one family. Autistic, social and psychiatric behaviors were also observed in one family with ID and motor abnormalities; and epileptic seizures, which were treated with broad spectrum and different anti-epileptic drugs, including levetiracetam, carmazepin, and phenobarbital, were reported in four different families (Fig. 1, Table 1). Although most families presented with different phenotypic manifestations, all presented ID, and most of them featured some kind of motor abnormality.

**Table 1:** Detailed phenotypic manifestations of families with ID syndromes.

Family/Disease	Approaches	Gene (mutation)	Cognition	Movement	Seizure	Consanguinity	Other
ID-Fam01 (Sanfilippo syndrome)	**HM & WGS**	**Pts. II-1, - 2, -3** ***NAGLU*** **(p.D312N)**	Severe ID	No movement abnormalities	yes	Yes	—
ID-Fam02 (Renal glycosuria and ID)	**HM & WGS**	**Pts. II-1, -2, -3** ***SLC5A2*** **(p.C361Y)**	Moderate ID and dysarthria	Truncal ataxia and titubation, impaired finger to nose exam, increased tone in lower limbs and spasticity, increased deep tendon reflexes, upward plantar reflexes, and spastic-ataxic gait	Yes	Yes	Glucosuria
ID-Fam03 (Familial hypomagnesemia)	**HM & WGS**	**Pt. II-1** ***POLR3B*** **(p.V609M, p.V667M)**	ID from childhood and mental regression, anxiety and abnormal social behavioral disorders, pseudobulbar affect, and autistic-like symptoms	Stereotyped abnormal cervical movements, hyperreflexia (DTR: + 3), hesitate speech and stuttering, mild spastic muscle tone, babinski sign, terminal dysmetry and abnormal tandem gait, wide base spastic and ataxic gait with flexed arms on elbow and fisting	Yes	Yes	Bilateral cataract, basal ganglia calcification, mild hydrocephaly and generalized and cerebellar atrophy
		**Pt. II-2** ***POLR3B*** **(p.V609M, p.V667M)**	ID from childhood and mental regression ADHD, OCD (washing) and aggressive disorders	Mild Spastic muscle tone, hyperreflexia (DTR: + 3), babinski sign, terminal dysmetry and abnormal tandem gait, wide base spastic and ataxic gait with flexed arms on elbow and fisting	No	Yes	Bilateral cataract, basal ganglia calcification, mild hydrocephaly and generalized and cerebellar atrophy
ID-Fam04 (Chorea-acanthocytosis)	**WGS**	**Pt. II-1** ***VPS13A*** **(p.R1922X, p.R1961X)**	Mental regression and impaired recent memory	Oromandibular dyskinesia, choreaic movement of tongue that interfere with eating and speaking, rubber man like body movement and some motor tics, dystonic gait with bilateral foot drop	Yes	Yes	Generalized atrophy in brain MRI
ID-Fam05 (Autism and progressive ID without epilepsy) (3 male patients)	**HM & WGS**	**Pts. II-4, -5** ***SYN1*** **(p.R420Q)**	ID from early childhood mental regression Autistic features	No marked rigidity or tremor	No	No	Abnormal eye contact and language problem, sphincter dysfunction, marked generalized frontal atrophy in brain MRI
ID-Fam06 (HSP and mild intellectual disability)	**HM & WGS**	**Pt. II-1** ***SPG11*** (p.M245Vfs*2)	Mild ID	Resting tremor that aggravated with action and intention, severe spastic gait with some knee and ankle contracture, hyperreflexia with bilateral, babinski sign, mild bilateral dysmetry in finger to nose test	No	Yes	Typical thinning of corpus callusom and “ear of the lynx” appearance in anterior, aspect of ventriculs on MRI, bilateral jerk gaze evoked horizontal nystagmus, swan neck deformity in fingers and mild hyper-laxity of phalanx, discoloration of frontal skin to a blue-gray color
		**Pt. II-2** ***SPG11*** (p.M245Vfs*2)	Mild ID	Spastic gait with severe hyperreflexia and Babinski sign, mild bilateral dysmetry in finger to nose test	No	Yes	Discoloration of frontal skin to a blue-gray color Typical thinning of corpus callusom and “ear of the lynx” appearance in anterior aspect of ventriculs on MRI
		**Pt. II-3** ***SPG11*** (p.M245Vfs*2)	Mild ID	Hyperreflexia and Babinski sign, mild terminal dysmetry in finger to nose test, resting tremor, spastic gait	No	Yes	Hyperlaxity in phalanx with swan neck appearance, blue-gray discoloration of skin (especially frontal), typical thinning of corpus callusom and “ear of the lynx” appearance in anterior aspect of ventriculus on MRI
ID-Fam07 (HSP and mild intellectual disability)	**HM & WGS**	**Pt. II-1/Pt. II-2** ***NSL1*** **(p.R74P)**	Mild ID	Spastic paraparesis with hyperreflexia, Babinski sign and spastic gait	Yes	Yes	gradually decreasing vision

ADHD: Attention deficit hyperactivity disorder; OCD: Obsessive-compulsive disorder.

### Genetic findings

**Figure 2:**
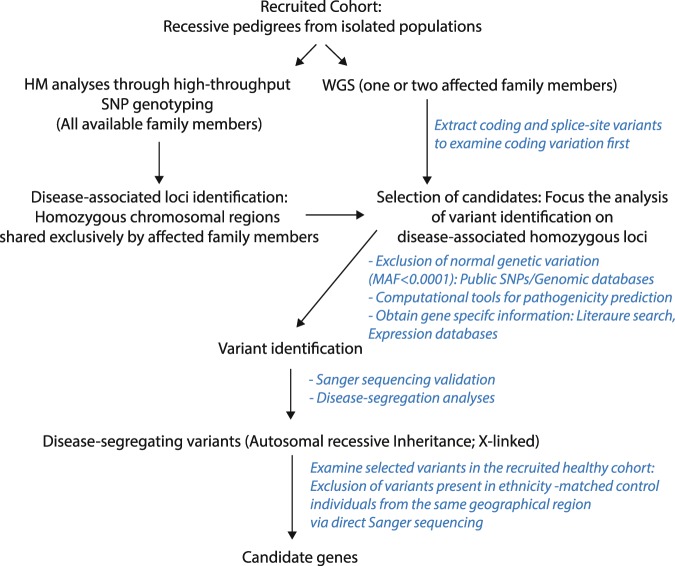
Flow chart for WGS analysis. It shows the steps carried out in the recruited DNA samples for accurate and reliable disease-gene identification.

Because the performed HM analyses identified several disease-associated loci in each individual family (Table 2), WGS analyses (Fig. 2) were then carried out in one (Family 7) or two (the remaining families) affected members from each individual family. These analyses led to the identification of several rare coding variations (Table 2), but only rare genetic variations within the associated loci were considered further (Fig. 2). Six out of seven families were found to carry pathogenic mutations in known NDD genes, with four mutations already reported and known to be associated with clinical phenotypes similar to those observed in our recruited families; while the two other, located in *SLC5A2* (solute carrier family 5 member 2) and *SYN1* (synapsin 1) genes, were novel and found to be absent in ethnicity-matched neurological individuals and public databases (see WGS methods for more details). Subsequent *Sanger* sequencing analyses revealed that all identified mutations segregated with the disease status.

**Table 2:** Homozygous segments identified to be shared exclusively by affected family members and genetic variation identified in the patients’ WGS data.

Family (ID)/Patients		HM data	Genetic variation: MAF <0.0001, <0.001 (WGS data)					
		Homozygous tracks (n°)	Non-synonymous	Stop-gained	Start-loss	Stop-loss	Frameshift	Splice-site
Fam ID-01	Patient II-1	8	589	20	1	0	138	59
	Patient II-2		613	19	5	2	148	67
Fam ID-02	Patient II-1	2	718	23	1	3	197	70
	Patient II-3		742	17	1	4	193	72
Fam ID-03	Patient II-1	12	667	13	3	2	146	81
	Patient II-2		663	16	3	2	148	68
Fam ID-04	Patient II-1	NA	563	17	1	2	124	58
Fam ID-05	Patient II-4	11	644	20	2	1	156	58
	Patient II-5		701	21	3	3	218	61
Fam ID-06	Patient II-1	12	687	20	3	3	154	71
	Patient II-2		660	22	2	3	157	61
Fam ID-07	Patient II-1	18	702	18	5	4	132	70
	Patient II-2		N.A	N.A	N.A	N.A	N.A	N.A

The affected subjects from Family Fam-01, who presented with severe ID and epilepsy but without behavioral abnormalities, were found to carry a known pathogenic mutation (p.Asp312Asn) in the *NAGLU* (N-acetyl-alpha-glucosaminidase) gene. This mutation was previously reported in patients with Sanfilippo syndrome B or mucopolysaccharidosis type III (MPS III) – a lysosomal storage disease characterized by behavioral changes including hyperactivity, aggressiveness, and destructive behaviors that progress to profound cognitive impairment and severe disability^8^ – and in a patient with severe ID^9^. Patients from family Fam-02, who presented with ID and motor abnormalities, were found to carry a novel mutation (p.Cys361Tyr) in the *SLC5A2* gene, also known as sodium-glucose transporter 2 (*SGLT2*). Mutations in *SLC5A2* are known to cause renal glycosuria^10^, which was also a symptom found in our patients with *SLC5A2* mutations. This mutation was absent in 182 ethnicity-matched control chromosomes and public databases and was predicted to be pathogenic by all the computational methods used, including Mutation Taster (disease-causing), MutPred (0.842), SIFT (damage), SNPs&Go (disease-causing; 0.837), and CADD (25.5). Patients from family Fam-03 manifested a complex clinical phenotype consisting of ID, severe behavioral problems, and movement abnormalities, including ataxia and spasticity among others, and were found to carry a known pathogenic mutation (p.Val667Met) in the *POLR3B* (RNA polymerase III subunit B) gene. *POLR3B* genetic variations are responsible for hypomyelinating leukodystrophy-8 that manifested with similar clinical symptoms as those observed in our family^11^. The only affected member from family Fam-04 was found to carry a known pathogenic mutation (p.Arg1922/1961Stop) in the vacuolar protein sorting 13 homolog A (*VPS13A*) gene, mutations of which are responsible for choreoacanthocytosis (CHAC)^12^. During the disease segregation analyses, an additional sibling (II-2) in this family, who remains unaffected at 30 years old, was found to carry the identified homozygous *VPS13A* mutation. The clinical features of our patient resemble those described in other reported CHAC patients. Both available patients from family Fam-05 were found to share 11 small tracks of homozygosity (~1Kb-1.5Kb), in which no homozygous genetic variations, including single nucleotide variations (SNVs) or indels, were identified. No compound heterozygous mutations were identified in both affected subjects. By contrast, a novel hemizygous mutation was identified in the *SYN1* gene (Xp11.3-p11.23), which encodes for synapsin-1 and has been associated with ASD and epileptic syndromes^13,14^. This novel *SYN1* missense genetic variation, which consisted of a c.1259 G > A transition that resulted in p.Arg420Gln amino-acid substitution, was found to be highly conserved among other species and absent in 840 ethnicity-matched control chromosomes and public databases. It was additionally predicted to be pathogenic by several computational programs including Mutation Taster (disease-causing), MutPred (0.568), and CADD (15.73). Our patients with this novel *SYN1* mutation presented with ASD-like features and ID without epileptic seizures. The three affected members from Fam-06 were found to carry a known and homozygous pathogenic mutation (p.Met245Valfs*2) in the *SPG11* gene, which is the most commonly mutated gene in complex autosomal recessive hereditary spastic paraplegia (AR-HSP), which presents with spastic paraplegia along with other clinical manifestations including ID. Our patients presented with clinical features similar to those found in AR-HSP including the presence of thin corpus callosum^15,16^.

**Figure 3:**
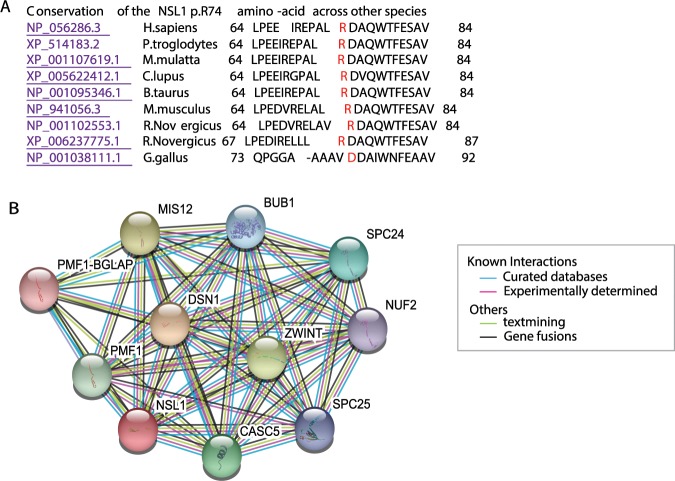
(**A**) Conservation of the amino-acid arginine at position 74 of the NSL1 protein across other orthologous. (**B**) Known and predicted protein-interactions of NSL1 protein according to STRING database. Kinetochore proteins that act as components of the essential kinetochore- associated NDC80 complex, which is required for chromosome segregation and spindle checkpoint activity: **NUF2, SPC24**, and **SPC25**. Kinetochore proteins that are part of the MIS12 complex, which is required for normal chromosome alignment and segregation and for kinetochore formation during mitosis: **MIS12, PMF1-BGLAP** (Polyamine-modulated factor 1), **DSN1** (Kinetochore-associated protein **DSN1** homolog), NSL1 (Kinetochore-associated protein NSL1 homolog). Kinetochore proteins that are essential for spindle-assembly checkpoint signaling and for correct chromosome alignment: **CASC5** (KNL1; Kinetochore scaffold 1) and **BUB1** (Mitotic checkpoint serine/threonine-protein kinase BUB1).

Lastly, only one family (Family 7) was identified with pathogenic mutations in an unknown disease gene. In this family only patient 1 was subjected to WGS analyses. The patient’s WGS data identified 12 different homozygous missense single-nucleotide variations (SNVs), with two of them located within previously associated homozygous segments. No compound heterozygous variation was identified. These two SNVs were located within *NSL1* (p.Arg74Pro) and *RTL1* (p.Arg235His) genes. Both *RTL1* (retrotransposon-like protein 1) and *NSL1* (Kinetochore-associated protein NSL1 homolog that is a component of MIS12 kinetochore complex) mutations were validated in the patient 2, who was a homozygous carrier for both mutations, and both were found to segregate with disease status. The *RTL1* genetic variation was found in heterozygosis in both healthy parents and a healthy sibling and found to be not present in an additional healthy sibling. The healthy family members were found to be either heterozygous carriers (n = 3) or non-carriers (n = 1) for the *NSL1* genetic variation (Fig. 1). The *RTL1* p.Arg275His mutation was not present in either the Iranome or GME variome databases but it is described in the gnomAD database with a frequency of 2.7e-5 (rs926337313) and it was found in homozygosis in two out of the 91 ethnicity-matched control subjects screened by us through *Sanger* sequencing. The *NSL1* p.Arg74Pro mutation is novel and found to be not present in any of the public SNP databases or in any of ethnicity-matched control subjects investigated through *Sanger* sequencing (n = 91). In addition, the *NSL1* p.Arg74Pro mutation with two mutated nucleotides (Fig. 1), affecting a highly conserved amino-acid (Fig. 3A), was predicted pathogenic by various computational methods [Mutation Taster (disease-causing), MutPred (0.624), SIFT (damage), and CADD (14.58)], while the *RTL1* mutation was predicted non-pathogenic by most of them [Mutation Taster (polymorphism), MutPred (0.488), and CADD (9.858)]. Both *RTL1* and *NSL1* genes are expressed in brain tissues, with *RTL1* showing lower brain expression, being localized to the substantia nigra and hypothalamus, while *NSL1* was found to be more ubiquitously expressed at higher levels in brain and other tissues, with its expression being higher in the spinal cord (https://gtexportal.org/home/). Given the role of kinetochores in neuronal development^17^, we also searched for protein-protein interactions in the STRING database to explore whether any of the kinetochore-related proteins associated with ID and microcephaly syndromes interacts with NSL1 and found that NSL1 interact with KNL1 (Fig. 3B).

### Functional results

#### Reduced neurite outgrowth was observed in mutant SYN1 cells

**Figure 4:**
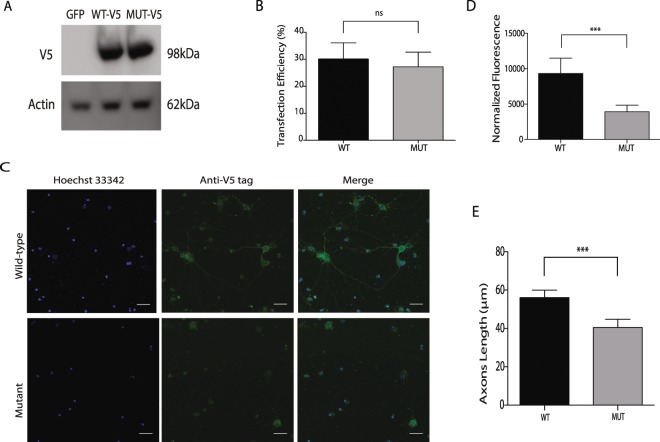
Effects of *SYN1* R420G mutation on hippocampal neurons. (**A**) Western blot in HEK293T cells untransfected or transfected with either wild-type of R420G SYN1-V5 mutant confirm expression of both constructs. Original western blot images are provided as Supplementary Material (**B**) Transfection efficiency on hippocampal neurons was calculated in three independent experiments by counting GFP+ cells. Black solid column shows wild-type SYN1-V5 transfected neurons and solid grey column shows R420G SYN1-V5 mutant transfected neurons **C:** Microscopy images of hippocampal neurons transfected with either wild-type (upper panel) or mutant (lower panel) SYN1-V5 (Scale bar: 25μM). (**D**) Neurons transfected with wild-type SYN1-V5 express higher levels of SYN1 (*p* = *0.0004*) and (**E**) have longer axons (*p* = *0.0008*) than their mutant counterparts. The length of axons and normalized fluorescence were measured using the ImageJ software. The graphs shows the mean normalize fluoresce (upper graph) and the axon length in μM (lower graph) of three independent transfections. Black columns show wild-type SYN1-V5 transfected neurons and solid grey columns show R420G SYN1-V5 mutant transfected neurons. Values represent the means ± SEM. ***p < *0.001*; ns = non-significant.

One of the families reported here was shown to have an X-linked disease due to a novel *SYN1* mutation. To further assess the deleterious effects of the disease-segregating *SYN1* mutation, primary hippocampal neurons were transfected and allowed to express either wild-type (wt) or mutant (m) SYN1 protein (Fig. 4A). The transfected cells were identified by the expression of GFP. A similar transfection efficiency (~30%) was observed between wt and m proteins (Fig. 4B). However, the mutant primary hippocampal neurons showed lower expression levels than wt neurons (*P* = 0.0004), indicating that the mutant SYN1 protein led to a significantly reduced neurite outgrowth (Fig. 4C,D).

#### Neurite development is impaired in mutant syn1 hippocampal neurons

Synapsins are well known for their participation in the developing nervous system. Particularly, both SYN1 and SYN2 expressions are increased during the formation of neurites, specification of axons, and synapse formation in mammals^18,19^, and both are involved in neuronal development, axonogenesis and synaptogenesis^20,21^, Thus, we examined and analyzed the neurites of syn1 wt and m cells. We observed that the length of the neurites in the mutant syn1 cells was shorter than that observed in their wild-type counterparts and that this difference was statistically significant (Fig. 4C,E).

## Discussion

In this study, we reported the clinical features and genetic findings of seven different families suffering from complex NDD syndromes. Taking into consideration the complexity of the genetic architecture and the phenotypic heterogeneity of the NDDs^22^, we used a combination of HM and WGS approaches to establish the disease-causing genetic variations in the affected pedigrees. My group and others have repeatedly demonstrated how powerful the combination of these two techniques is in identifying disease-associated genetic variations and genes^23–30^. All families were found to carry pathogenic genetic variations in different disease genes, with six families having genetic variations (4 known and 2 novel) in known genes, while one family carried a pathogenic genetic variation in a novel gene (Table 1). The two novel genetic variations identified in known disease genes, *SLC5A2* and *SYN1*, are likely to be pathogenic, as they were the only disease-segregating mutations identified within the disease-associated loci, and both were absent in ethnicity-matched neurologically normal individuals and public SNP databases. Further, our examined patients presented with similar phenotypic manifestations to those previously reported, including the presence of renal glycosuria in patients with *SLC5A2* mutations. Developmental delay and movement abnormalities have also been reported in patients with renal glycosuria and *SLC5A2* mutations^10^. Although seizures were not observed in our patients with *SYN1* mutations, *SYN1* mutations have been found in individuals with epilepsy, ASD, or both. The mutated amino acid in SYN1 (420aa) is located very close to the Synapsin_C domain (214–416 aa), which is an ATP binding domain that is highly conserved across all the synapsins and is a feature of all splice variants. We, therefore, examined the functional effects of the mutant SYN1 protein by mutating the affected amino acid in hippocampal neurons: we observed significantly reduced neurite outgrowth and impaired axonal development in mutant cells when compared to wild-type counterparts (Fig. 4), thus, supporting its pathogenic effect on the protein function and confirming the role of SYN1 function in neuronal development and axonal outgrowth.

Among the pedigrees examined here, we identified one with a pathogenic genetic variation in a novel gene not previously associated with ID syndromes. In this family, which presented with ID, spastic paraplegia and visual impairments, two genetic variations within two different genes (*NSL1* and *RTL1*) were identified. The genetic variation within the *RTL1* gene was found in both public databases and ethnicity-matched control sequenced by us. Furthermore, the *RTL1* gene encodes for a retrotransposon-like protein 1, whereas *NSL1* encodes for a kinetochore-associated protein that contains two coiled-coil domains that localize to kinetochores, which are chromosome-associated structures that attach to microtubules and mediate chromosome movements during cell division. The kinetochores are broadly conserved from yeasts to humans and their constitutive proteins have an essential post-mitotic function in neuronal development^17^. Interestingly, mutations in microtubule-related genes, including *DYNC1H1* (dynein cytoplasmic 1 heavy chain 1), *KIF5C* (kinesin family member 5 C), *KIF2A* (kinesin family member 2 A), and *TUBG1* (tubulin gamma 1) have been shown to cause malformations of cortical development associated with severe ID and/or microcephaly^31^; *de-novo* truncating mutations in *CHAMP1* (chromosome alignment maintaining phosphoprotein 1), a protein that is involved in kinetochore-microtubule attachment, have been identified in patients with syndromic ID and dysmorphic facial features^32–34^; and mutations in *KNL1* (CASC5), which is a kinetochore scaffold 1 protein that is also required for creation of kinetochore-microtubule attachments and chromosome segregation, and interacts with NSL1 (Fig. 3), are a cause of autosomal recessive primary microcephaly-4 (MCPH4)^35^.

Taken together, the following evidence strongly supports the novel *NSL1* p.Arg74Pro mutation as the genetic variation responsible for causing disease in our reported family: (**1**) The role of kinetochore-associated proteins in neuronal development and their associations with ID and microcephaly syndromes, (**2**) the interaction of NSL1 with other kinetochore proteins responsible for causing ID (Fig. 3B), and (**3**) the fact that the novel *NSL1* p.Arg74Pro mutation is predicted highly pathogenic and is highly expressed in brain tissues. Therefore, we believe that impairments within the NSL1 kinetochore-protein might be associated with the development of complex ID and might affect the interaction with other ID-associated kinetochore proteins leading to the development of NDD.

Despite the fact that all the families presented with ID, the clinical spectrum was different in the majority of them (Table 1). All (n = 5) but two families presented with motor abnormalities along with the ID features. The high frequency of motor abnormalities in our families is not surprising since these and seizures are frequently observed in patients with ID^4^. Novel genes causing ID and parkinsonism have been also recently identified through both whole exome and genome analyses^24,36–38^, seizures have been reported in patients with parkinsonism^39,40^, and a high frequency of parkinsonism has been observed among adults with ASD^41^. Epileptic seizures were also present in families with *NAGLU*, *SLC5A2*, *POLR3B*, and *VPS13A* mutations (Table 1), and behavioral and psychiatric symptoms were observed in a family with POLR3B mutations (Fam 03). Epilepsy is relatively prevalent in people with ID, being more common in people with ID than in the general population, and its prevalence increases with the severity of disability^42^. Existing evidence suggest that epileptic seizures and side effects of antiepileptic drugs can directly damage individual’s cognitive ability and physical health through repeated head injury and seizures episodes. In fact, behavior abnormalities and psychiatric disorders occur significantly more often in people with epilepsy and people with ID than in “healthy” subjects^42,43^. Thus, it is not surprising that four out of the seven families reported here also feature epileptic seizures. To summarize, most of our patients with mutations in known genes presented with similar phenotypic manifestations to those previously reported (Table 1), with the exception of our patients with the *NAGLU* p.D312N mutation, who did not show any behavioral symptom.

In conclusion, we report the genetic defects, including the identification of a novel kinetochore gene as responsible for ID, and the phenotypic manifestations of seven different families with complex ID syndromes, most of which presented with other comorbidities, including epilepsy, motor abnormalities, psychiatric symptoms, and ASD. Given the fact that the neurodevelopmental disorders can be attributed to mutations at over 1000 loci, it is not surprising that different genes were identified to be mutated in each individual family^4^. Both the phenotypic and genetic heterogeneities associated with NDD disorders make their differential diagnosis increasingly complex^44^, and thus, we believe that the combination of HM and WGS is a suitable and fruitful strategy to identify the gene defects in recessive pedigrees with parental consanguinity, as it allows to target genomic regions for the analysis by WGS of a single-family member and to determine if distinct causes are responsible for the co-occurrences of rare phenotypes in the same pedigree.

## Materials and Methods

### Subjects

Seven different families from the Middle East and featuring complex NDD syndromes were clinically diagnosed and subjected to both HM and WGS analyses. The Wechsler IQ test was used to classify the severity of ID. Six out of seven families were consanguineous with parents being first cousins. 420 DNA samples belonging to ethnicity-matched control subjects from different ethnic populations living in Iran (Arabs, Azeris, Balochs, Kurds, Lurs, Persians) were also available for study. DNA samples from all members were isolated from whole blood using standard procedures. The local ethics committee at the Semnan University of Medical Sciences approved this study, and informed consent according to the Declaration of Helsinki was obtained from all participants.

### Genetic approaches

Due to the phenotypic heterogeneity observed in our recruited pedigrees (Table 1) and the parental consanguinity observed in six of them (Fig. 1), both HM and WGS analyses were performed to facilitate the identification of the disease-associated mutations. HM analyses were performed in all the families consisting of more than one affected member (n = 6), while the disease-associated mutation for family Fam-04 that consisted of one affected patient was exclusively determined through WGS analysis.

#### Homozygosity mapping (HM)

High throughput SNP genotyping was carried out in all available family members of six different families (n = 34) (Fig. 1A) using the HumanOmniExpress Exome arrays v1.3 and the HiScanSQ system (Illumina Inc., San Diego, CA, USA). The GenomeStudio program (GS; Illumina) was used to undertake quality assessments and generate PLINK input reports for HM^45^. Homozygous segments across all family members in each pedigree were determined as previously described, and only those shared by affected family members but not by healthy subjects were considered as disease-associated loci^24,27,29^. HM analyses were not carried out in Family ID-Fam04, in which direct WGS to identify the gene defects was performed in the only affected family member.

#### Whole genome sequencing (WGS)

WGS was carried out at the New York Genome Center (NYGC) in at least two affected family members from each family with the exception of family 04 and family 07 in which only one affected subject was subject to WGS analyses (n = 12). Briefly, sequencing libraries were constructed with the TruSeq PCR-free library kit (Illumina) following the manufacturer’s recommended protocol and sequenced on the Illumina HiSeq X instruments, with 2 × 150 bp paired reads, to a minimum coverage of > 30 × . Sequencing data was processed with NYGC’s automated analysis pipeline, which includes alignment to GRCh37 using BWA-MEM (v0.7.8)^46^, and further processing with GATK best practices, including the marking of duplicates with Picard (v1.83, http://picard.sourceforge.net) and GATK (v3.2.2)^47^. Single nucleotide variations (SNVs) and indels were called by using the GATK HaplotypeCaller and were jointly genotyped. Deletions were called by using GenomeSTRiP (v2.0)^48^ and were jointly called by using 17 HapMap individuals (CEPH Platinum Genomes pedigree). All deletions annotated as PASS in the GenomeSTRiP results were further filtered by using custom scripts to remove redundant calls and breakpoints overlapping repeat regions, or with extensive mapping ambiguity. Annotations of variants included predictions of the effect of nucleotide change on protein sequence using SnpEFF; variant frequencies in different populations from 1000 Genomes project, NHLBI GO Exome Sequencing Project; cross-species conservation scores from PhyloP, Genomic Evolutionary Rate Profiling (GERP), PhastCons; functional prediction scores from Polyphen2, SIFT, and CADD^49^; and variant disease associations from OMIM, Clinvar, Genetic Association Database (GAD); regulatory annotations from ENCODE, RegulomeDB, ORegAnno, KEGG pathway annotations; transcription factor binding sites from the Transfac database; and Gene Ontology (GO) annotations for biological process, cellular component, molecular function.

#### Selection of candidates: Identification of disease-causing variations

Because in recessive pedigrees, healthy parents are obligate heterozygous carriers for the mutant allele, only homozygous and compound-heterozygous genetic variations were considered as potential disease-causing mutations. The combination of HM and WGS analyses allowed us to further reduce the number of candidate genetic variations in each individual pedigree. Only the genetic variations within the disease-associated chromosomal regions were potentially considered as disease-associated mutations. Thus, only novel and rare (MAF <0.0001, <0.01%) non-synonymous missense, nonsense, frame-shift, gain or loss of stop codon genetic variations within the disease-associated loci were examined further (Fig. 2)^24,50,51^. To assist with gene identification, the frequency of selected candidates was examined in additional public databases, including the Iranome database, which contains whole exome data of 800 individuals from eight major ethnic groups in Iran (http://www.iranome.com/about), the Greater Middle-East variome that contains exome data from 2,497 individuals (http://igm.ucsd.edu/gme/), and the Genome Aggregation database that contains data from 125,748 exome sequences and 15,708 whole-genome sequences (gnomAD; https://gnomad.broadinstitute.org/). The pathogenicity of the novel identified mutations was predicted by two additional computational methods (MutPred and SNPs&GO) that have been evaluated as most efficient^52^ and were not included in the annotation files (Fig. 2).

#### Variant validation and disease segregation assays

*Sanger* sequencing using primers flanking the exons where the disease-associated mutations were located (*NAGLU* exon 5, *SLC5A2* exon 9, *POLR3B* exon 19, *VPS13A* exon 45*, SYN1* exon 10, *SPG11* exon 4, *NSL1* exon 1, and *RTL1* exon 1) was used to validate the identified mutations and to perform the disease segregation analyses. Primer sequences were designed by using a public primer design website (http://ihg.gsf.de/ihg/ExonPrimer.html) (Primers sequences available upon request). All purified PCR products were sequenced in both forward and reverse directions with Applied Biosystems BigDye Terminator v3.1 sequencing chemistry as per the manufacturer’s instructions, and resolved and analyzed as described elsewhere^27^.

### Functional assays

#### SYN1 *in-vitro* assays

The effects of the wild-type (wt) and mutant (m) amino-acids of a novel *SYN1* mutation (p.Arg420Gln) identified in a patient with ID and autistic-like features were examined through *in-vitro* assays. The *SYN1* gene encodes two different isoforms: the isoform SYN1a (NP_598006.1) with 705 amino acids and the isoform SYN1b (NP_008881.2) containing 669 amino acids. Both isoforms are exclusively expressed in brain tissues, with transcript SYN1a expressed at higher levels.

For site-directed mutagenesis and cloning, The pANT7_cGST (GST-tagged *in vitro* expression vector) SYN1b (Homo sapiens) plasmid was obtained from DNASU Plasmid Repository (Clone ID HsCD00639921) and deposited from the Center for Personalized Diagnostics at Arizona State University (http://dnasu.org/)^53^. The plasmid was expanded using a Qiagen Spin MiniPrep Kit (Qiagen, Germany). Once validated, a mutation affecting the 420 amino-acid (p.R420G) was introduced by using the QuickChange II XL Site Directed Mutagenesis Kit (Agilent Technologies, USA) following the manufacturer’s recommendations. Primers used to generate the mutation were designed by using QuickChange Primer Design Tool from Agilent Technologies, and primers used to verify the mutation were designed using Primer 3 program (http://bioinfo.ut.ee/primer3-0.4.0/). All constructs were verified in both forward and reverse directions through *Sanger* sequencing. Subsequently, SYN1 wt and m cDNA in pANT7_cGST-SYN1 plasmid were amplified using corresponding primers (primer sequences available upon request). To generate the longer SYN1a transcript, two different pairs of primers were designed and used to amplify two PCR products containing respectively the 669 amino-acids of SYN1b and the 36 missing amino-acids of SYN1a. Both PCR fragments were then joined and amplified by overlapping PCR, sub-cloned into pcDNA3.1V5/HisA vector (Clontech, Mountain View, CA, USA) between EcoR1 and Xba1 sites, and verified by *Sanger* sequencing. Their expressions were confirmed by transfecting them into HEK293T cells and immunoblotting the lysates with anti-V5 antibody (ThermoFisher, R960-25).

For cell transfections, HEK293T were maintained in 1X Dubelcco’s Modified Eagle’s Medium (1x DMEM) (Corning, USA), supplemented with 10% Fetal Bovine Serum (FBS) and 1% Penicillin/Streptomycin (GIBCO, USA), and kept in a 37 °C/5%CO2 incubator. Primary hippocampal neuron cultures were prepared from E15.5 wt C57BL6 mouse embryo as previously described^54^, maintained in neurobasal medium supplemented with B27 and 1% Penicillin/Streptomycin, and kept in a 37 °C/5%CO2 incubator. Both HEK293T cells and hippocampal neurons were plated in 24-well plates with a density around 2*10E05 cells/ml and transfected by using Lipofectamine 3000 (ThemoFisher, USA) according to the manufacturer’s instructions. Briefly, 1 µg of plasmid and 3 µl of Lipofectamine separately diluted in 100 µl of Opti-MEM (GIBCO, USA) were mixed and incubated for 15 min. The 200 µl of plasmid:lipofectamine complex was then added to cells drop by drop and mixed by gently agitation. Transfection efficiency was measured by the number of cells expressing green fluorescent protein (GFP) compared to the total number of cells.

#### Immunocytochemistry

Non-transfected and transfected hippocampal neurons with either wt or m plasmid were plated on poly-D-lysine coated coverslips at a density of 100/mm^2^ in a 6-well plate, cultured for 14 days, and fixed by 4% paraformaldehyde treatment. For immunostaining cells were permeabilized with 0.05% Triton X-100 (Sigma Aldrich, USA) for 10 min, blocked with 1% BSA for 30 min, and incubated for 1 hour at room temperature (RT) with primary antibody Anti-V5 tag diluted 1:500 in 1% BSA (Thermo Fisher, USA, R960-25). After washing, fluorophore-conjugated secondary antibody was added for 1 hour at RT (anti-mouse 1:1000 in 1% BSA) (Jackson Immunoresearch, USA, 115-035-166). Cell nuclei were labeled using Hoechst 33342, Trihydrochloride, Trihydrate at 2μg/ml (Thermo Fisher, USA, H3570). Coverslips were mounted into the slides using Fluoromount-G™ Slide Mounting Medium (Electron Microscopy Sciences, USA).

#### Imaging

Immunolabeled hippocampal neurons were visualized by using a Leica SP5 DM microscope with a 63x magnification objective and analyzed through the ImageJ software (imagej.nih.gov). Confocal z-stack images were acquired in 17 (wt) and 14 (m) random locations within the coverslips of three independent transfections. For *SYN1* quantification, individual cells (42 wt and 44 m cells) were circled based on their fluorescent signals and the area and integrated mean intensity was then calculated in ImageJ. The corrected total cell fluorescence (CTCF) was calculated as Integrated Density (area of selected cells x mean fluorescence of background readings)^55^. The length of the neurites was measured by using the ImageJ software as described elsewhere^13^.

#### Western blot

HEK293T cell lysates were collected using the radioactive immunoprecipitation assay (RIPA) buffer (Sigma) with a phosphatase inhibitor (Roche). HEK293T cell lysates were loaded onto NuPAGE 4–12% Bis-Tris Protein Gel (Thermo Fisher, NP0336BOX) and resolved in 1x NuPAGE MES SDS running buffer (Thermo Fisher, NP0002). Proteins were then transferred to PVDF membrane (Thermo Fisher, LC2002) using 1x transfer buffer containing 20% methanol. The membrane was blocked with 10% non-fat dry milk (LabScientific, 732-291-1940) in 1x Tris buffered saline containing 0.05% Tween (TBST) for 30 min and incubated with primary antibody against V5 (1:1000, Thermo Fisher, R960-25) overnight. The membrane was washed three times with 1x TBS, once with 10% non-fat dry milk, and incubated with horseradish peroxidase conjugated anti-mouse secondary antibody (1:5000, Jackson Immunoresearch, 115-035-166) for 30 min. The membrane was washed three times with 1 s TBST and chemiluminescent signal was developed by incubating the membrane with HRP substrate (SuperSignal West Dura, Thermo Fisher, 34075) and detected by G:BOX Chemi image analyzer (Syngene, USA). The membrane was stripped of anti-V5 antibody by incubating with 50 mM Glycine buffer, pH 2.2 for 30 min, and immunoblot procedure was repeated with anti-actin (1:1000, Sigma, A2066) and HRP conjugated anti-rabbit (1:5000, Jackson ImmunoResearch, 711-035-152) antibodies as described above.

#### Statistical analyses

Statistical analyses were performed using the Mann-Whitney nonparametric test in the GraphPad Prism software version 6.0 (GraphPad, USA). Data on graphs are presented as mean ± SEM. ***P < 0001; ns = non-significant.

## Supplementary information

Supplementary Material.

## Data Availability

The raw data that support the findings of this study are now available from the corresponding author upon reasonable request, and will be deposited in dbGAP upon publication.
