# Supplementary material for: Phenotypic and genotypic characterization of families with complex intellectual disability identified pathogenic genetic variations in known and novel disease genes
Source: Sci Rep. 2020 Jan 22;10:968. doi: 10.1038/s41598-020-57929-4 (PMC6976666; doi:10.1038/s41598-020-57929-4)
Supplement: Supplementary file 1 — Supplementary Material. [file 41598_2020_57929_MOESM1_ESM.pdf]

Supplementary Material: Original Western Blot Images From Figue 4B

Article Title: Phenotypic and genotypic characterization of families with complex intellectual disability identified pathogenic genetic variations in known and novel disease genes

Authors: Hossein Darvish, Luis J. Azcona, Abbas Tafakhori, Roxana Mesias, Elena Sanchez, Elham Alehabib, Azadeh Ahmadifard, Arman Habibi, Amir Hossein Johari, Babak Emamalizadeh, Faezeh Jamali, Marjan Chapi, Javad Jamshidi, Yuji Kajiwara, Coro Paisán-Ruiz

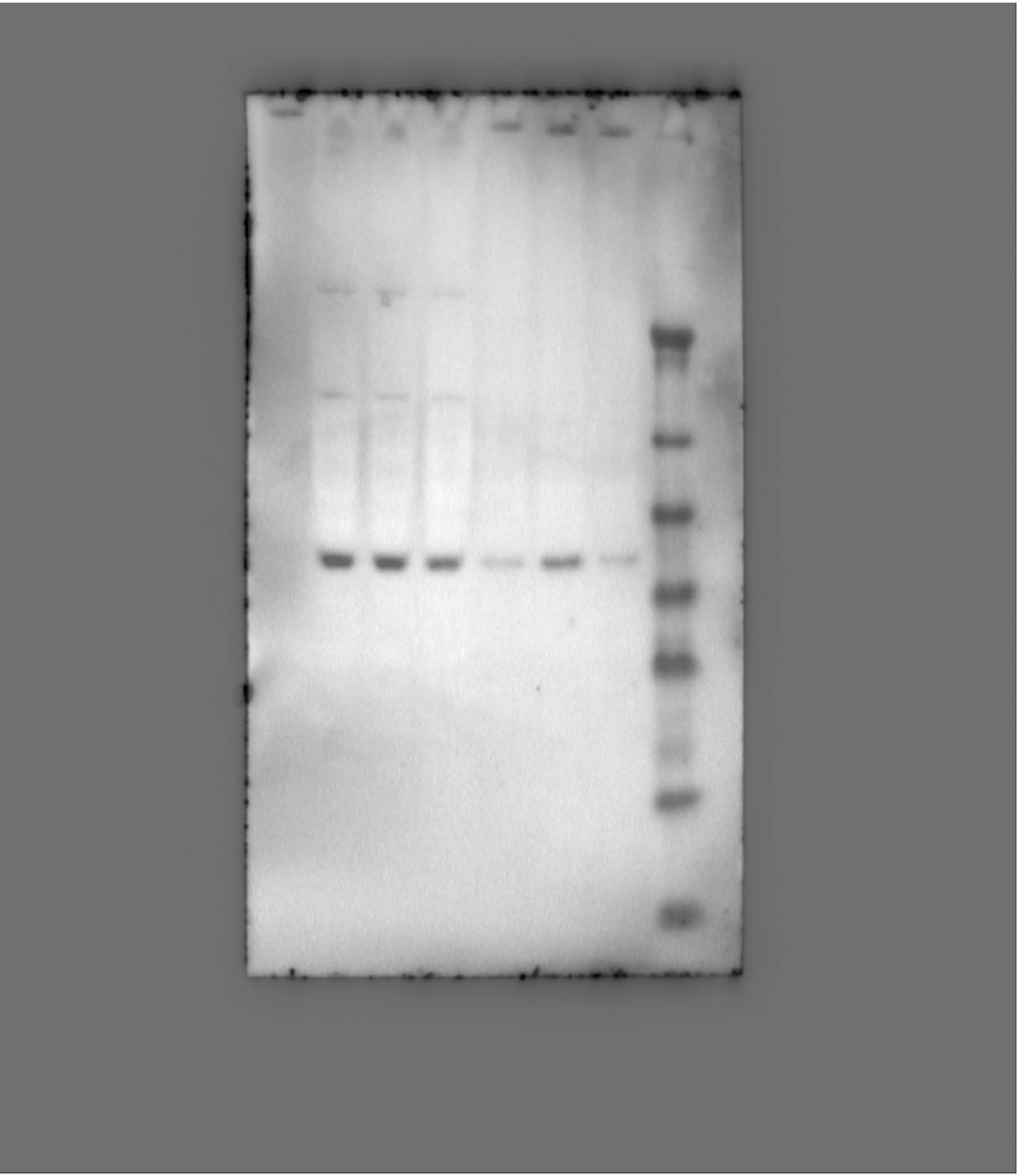

Original Western Blot Images From Figure 4B

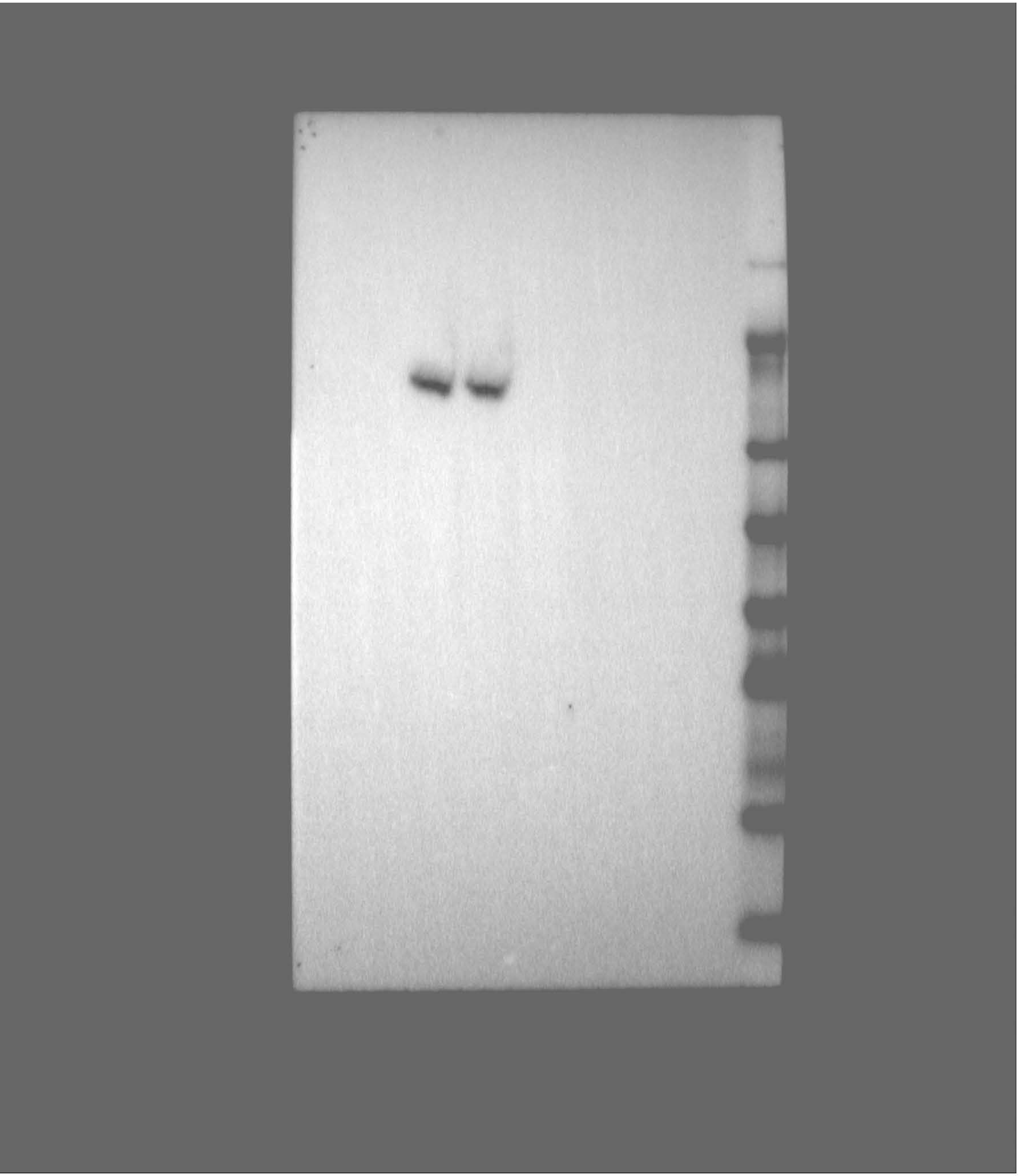

Original Western Blot Images From Figure 4B
